# Associations of the PREVENT Score with coronary artery and thoracic aortic calcification among South Asian Americans

**DOI:** 10.1016/j.ajpc.2026.101538

**Published:** 2026-03-11

**Authors:** Eve M. Manghis, Rachel S. Chang, Feng Lin, Meghana D. Gadgil, Nilay S. Shah, Matthew Budoff, Alka M. Kanaya

**Affiliations:** a Division of General Internal Medicine, Department of Medicine, University of California, 1545 Divisadero Street, San Francisco, CA 94143, USA; b Division of Cardiology, Department of Medicine, Northwestern University Feinberg School of Medicine, Chicago, IL. 676 N. St. Clair Street, Suite 600, Chicago, IL 60611, USA; c Division of Cardiology, Harbor-UCLA and The Lundquist Institute, 1124 W Carson St, Torrance, CA 90502, USA

**Keywords:** Subclinical atherosclerosis, Atherosclerosis, Coronary artery calcification, Thoracic aortic calcification, Risk stratification

## Abstract

**Background:**

South Asian populations have a high risk of atherosclerotic cardiovascular disease (ASCVD). Coronary artery calcification (CAC) can guide clinical decision making in patients with intermediate ASCVD risk. Thoracic aortic calcification (TAC) can be measured from the same computed tomography scan, though it is unknown whether TAC may help reclassify ASCVD risk.

**Methods:**

We conducted a cross-sectional analysis of the Mediators of Atherosclerosis in South Asians Living in America (MASALA) study. Ten-year ASCVD risk was calculated using the American Heart Association Predicting Risk of cardiovascular disease Events equation (PREVENT), categorized as <5 %, 5 to <10 % or ≥10 %. TAC and CAC scores were categorized as 0, 1-99, and ≥100. We determined associations of traditional risk factors with TAC and CAC and used cubic spline functions to examine non-linear associations between PREVENT and TAC and CAC, overall and stratified by gender.

**Results:**

Of 960 participants, 59.4 % had any CAC, 53.8 % had any TAC, and 42.4 % had both CAC and TAC. Any TAC and any CAC were each more likely with older age, among men, and in those with hypertension, diabetes, higher LDL-cholesterol, and statin use. Higher PREVENT-ASCVD risk was associated with higher TAC in women and higher CAC in men. Among women, TAC was significantly higher than CAC in PREVENT scores ≥7 %.

**Conclusion:**

In South Asian US adults, higher PREVENT-ASCVD risk is associated with higher CAC and TAC. TAC burden is higher than CAC among women with PREVENT scores 5 to <10 %. Further studies should explore if high TAC is predictive of clinical events.

## Highlights

• Thoracic aortic calcification (TAC) is a marker of subclinical atherosclerosis that can be obtained from the same computed tomography scan as coronary artery calcium (CAC).
• We report the association of ten-year ASCVD risk using the American Heart Association Predicting Risk of cardiovascular disease Events equation (PREVENT) equation with CAC and TAC.
• Higher PREVENT-ASCVD estimated risk was associated with higher TAC in women and higher CAC in men. Among women, TAC was significantly higher than CAC in PREVENT-ASCVD estimated risk scores exceeding 7 %.
• These findings suggest the potential utility of TAC potential utility of TAC as a tool for identifying subclinical atherosclerosis in women.

## 1 Introduction

South Asians are a large ancestral population group and are disproportionately affected by early and aggressive atherosclerotic cardiovascular disease (ASCVD). Globally, South Asians account for 60 % of heart disease and experience significantly higher mortality from heart disease compared to other population groups [[1], [2], [3], [4]]. Therefore, predicting and preventing ASCVD in this population is critical.

For many adults, primary ASCVD prevention includes calculating one’s estimated risk based on a population-based risk calculator. In 2024, the AHA introduced the Predicting Risk of CVD Events (PREVENT) equations [5,6]. PREVENT enables risk estimation in a broader age range (30-79 years), separately calculates risk of ASCVD, CVD, and heart failure, and provides different equations for 10- and 30-year risk. PREVENT additionally incorporates estimated glomerular filtration rate (eGFR), body mass index (BMI) and three optional predictors (hemoglobin A1c, urine albumin-creatinine ratio, and zip-code based social deprivation index) in risk equations and removes race as a risk variable [5].

Since 2018, the ACC/AHA guidelines for cholesterol management have recommended considering a coronary artery calcium (CAC) scan to guide clinical decision making for intermediate risk individuals [[7], [8], [9]]. Thoracic aortic calcification (TAC) is the most common form of extra-coronary artery calcium and can be measured from the same computed tomography (CT) scan as for measuring CAC, though it is unknown whether TAC may help in reclassification of ASCVD risk, or provides more risk information beyond CAC [10,11]. The association between traditional cardiovascular disease risk factors and the PREVENT scores with subclinical measures of atherosclerosis among South Asian Americans has not been reported previously.

Using data from the Mediators of Atherosclerosis in South Asians Living in America (MASALA) study, we aimed to investigate the association of traditional cardiovascular risk factors as well as PREVENT risk scores with prevalent TAC and CAC. Understanding this relationship may inform risk classification and tailor prevention strategies in this understudied population.

## 2 Methods

### 2.1 Study population

We utilized data from South Asian participants aged 40-84 years without prior clinical cardiovascular disease who participated in the Mediators of Atherosclerosis in South Asians Living in America (MASALA) study. MASALA is a longitudinal, community-based cohort study of South Asian men and women in the San Francisco Bay Area and greater Chicago area. Study methods have been published previously [12]. Institutional review boards at Northwestern University and the University of California, San Francisco approved study protocols. All participants gave written informed consent.

Participants were enrolled in MASALA at two timepoints; an initial cohort was recruited and enrolled from 2010 to 2013 and a returned between September 2015 and March 2018 for Exam 2. During 2017-2018, a new wave of participants was invited to enroll in the study (n = 258) and completed similar data collection. During baseline examination, participants completed questionnaires regarding demographic information, socioeconomic status, tobacco use, and medical history. Trained bilingual research staff recorded participant weight, height, and measured seated blood pressure using standardized protocols at each exam. Lipoprotein(a) mass concentration measurements were collected using a latex-enhanced turbidimetric immunoassay (Denka Seikan, Tokyo, Japan) [13].

Of the 1,007 participants examined in MASALA between 2015-2018, we excluded 47 who did not complete CT scans for TAC and CAC measurements. The remaining 960 participants comprise the overall study population for this analysis. Of these 960 participants, we excluded participants older than age 79 years or those missing data for the PREVENT scores, leaving a total of 911 for the PREVENT risk score analysis.

#### 2.1.1 Predictor: PREVENT equation

Ten-year PREVENT-ASCVD risk was calculated using MASALA study data from 2015-2018 with the following variables: age, gender, total cholesterol, high-density lipoprotein cholesterol, systolic blood pressure, estimated glomerular filtration rate, diabetes, current smoking, anti-hypertensive medication, lipid-lowering medication. We also added Hba1c to the base PREVENT-ASCVD equation.

Participants self-reported age and gender. Participant weight was measured on a standard balance-beam scale or digital weighing scale. Height was measured using a stadiometer. BMI was calculated by weight in kg divided by height in meters [2]. Systolic blood pressure was obtained by averaging the last two of three blood pressure measurements at rest by trained clinical staff. Participants were considered to have diabetes if they met any of the following criteria: 1) fasting blood glucose ≥126 mg/dL, 2) 2-hour glucose ≥200 mg/dL, or 3) use of medication use for diabetes. Total cholesterol and high-density lipoprotein cholesterol was obtained from serum samples after a 12-hour fast; LDL was calculated. Insulin was measured by the sandwich immunoassay method (Roche Elecsys 2010, Roche Diagnostics, Indianapolis, IN) from fasting serum samples [14]. The homeostasis model assessment of insulin resistance (HOMA-IR) was used as a measure of insulin resistance and calculated as insulin (mIU/mL) × glucose (mmol/L)/22.5. HOMA-β was used to measure β-cell function and was calculated as [20 × Insulin_0_(μIU/ml)/ Glucose_0_(mmol/l)−3.5] [14,15].

A single-slice abdominal non-contrast CT image was used to measure visceral fat area, which was defined as pixels with the appropriate Hounsfield Unit (HU) range inside the visceral cavity. Non-contrast cardiac-gated CT images were used to quantify hepatic attenuation. Lower levels of hepatic attenuation in HU demonstrate higher degrees of liver fat [16]. Antihypertensive and lipid-lowering medication (HMG-coA reductase inhibitor, fibrate, or niacin) use was ascertained by medication inventory.

Based on calculated PREVENT-ASCVD equation, participants were stratified to the following groups: <5 %, 5 to <10 %, or ≥10 % for the 10-year risk for ASCVD. Prior papers have utilized the similar ordinal classification for PREVENT as used in the ASCVD Pooled Cohort Equation (PCE) [17]; our sample had a very low proportion of individuals with a PREVENT score ≥20 %. The 2025 ACC/AHA Guidelines for the Prevention, Detection, Evaluation and Management of High Blood Pressure in Adults recommends using a PREVENT threshold of 7.5 % for the initiation of blood pressure agents in adults with blood pressure >130/80 (prior guidelines utilized a threshold of >10 % for the PCE) [18,19]; the PREVENT risk thresholds for the lipid therapy have not been published yet.

#### 2.1.2 Outcomes: subclinical atherosclerosis measures

Certified technologists scanned all participants using a research CT protocol that has been reported previously [20]. All imaging studies were interpreted at the Los Angeles Biomedical Research institute at Harbor University of California Los Angeles by radiologists blinded to clinical information. The Agatston method was then utilized to determine the presence and extent of calcium in each coronary artery and the thoracic aorta [14]. Given the skewness of these measures, we categorized TAC and CAC scores as 0, 1-99, and ≥100, consistent with clinical guidelines and prior reports.

### 2.2 Statistical analysis

We categorized PREVENT score into three categories (<5 %, 5 to <10 %, and ≥10 %) and compared demographic, behavioral, clinical and metabolic characteristics across the three categories using ANOVA or Kruskal-Wallis tests for continuous variables and chi-squared tests for categorical variables.

Given skewness of the calcification measures, we calculated median of TAC and CAC by PREVENT score category. We also evaluated the relationship between traditional cardiovascular risk factors and TAC and CAC categories with ANOVA, chi-squared, and/or Kruskall-Wallis tests as appropriate, and used Tobit regression to account for left-censoring at 0. We examined the relative frequency of PREVENT <5 %, 5 to <10 % and ≥10 % with three categories of TAC and CAC (0, 1-99, and ≥100) to evaluate concordance between categories of risk score and calcification outcome.

Unadjusted restricted cubic spline functions were used to explore potential non-linear associations between PREVENT scores and TAC and CAC, overall and stratified by gender. These restricted cubic spline plots used continuous measures of median CAC or median TAC by PREVENT scores, limiting participant data within the 5^th^ to 95^th^ percentile to lessen the influence of outliers for the plots. We also tested for interactions by gender.

We used STATA 18.0 and SAS 9.4 for all statistical analyses. A p value of <0.05 was used as threshold to define statistical significance.

## 3 Results

In 960 MASALA participants in this study population, mean age was 59.5±8.6 years, 54.4 % were men, and 32.1 % were using a statin (Table 1). TAC and CAC were modestly correlated with each other (R = 0.27), and the correlation was higher among women (R = 0.44) than men (R = 0.23). The presence of any CAC was more common than any TAC (59.4 % had any CAC and 53.8 % had any TAC). Overall, 29.3 % of the sample had no TAC and no CAC, 11.4 % had TAC>0 but no CAC, 17.0 % had CAC>0 but no TAC, and 42.4 % of the sample had both detectable CAC and TAC. A greater proportion of men than women had co-existing CAC and TAC (52.9 % in men compared to 29.9 % in women) (Supplemental Table 1).

**Table 1:** Characteristics of MASALA study participants overall and stratified by PREVENT score, 2015-2018.

		PREVENT, 10-year ASCVD score			
	Overall	≤5%	5 to <10%	≥10%	*P* value
	(n= 960)	(n=507)	(N=268)	(N=136)	
Age, years	59.5 ± 8.6	54.4 ± 5.9	63.8 ± 6.3	70 ± 6.5	<.001
Men	522 (54.4)	200 (39.4)	180 (67.2)	104 (76.5)	<.001
Family Income <$40k	60 (24.5)	24 (20.5)	21 (29.6)	8 (22.9)	0.533
$40-75k	31 (12.7)	15 (12.8)	8 (11.3)	6 (17.1)	
$75-100k	23 (9.4)	12 (10.3)	4 (5.6)	5 (14.3)	
≥$100k	131 (53.5)	66 (56.4)	38 (53.5)	16 (45.7)	
Years lived in United States	30.1 (12.2)	26.6 (10.9)	33.3 (12.1)	36.9 (12.5)	<.001
Systolic BP, mmHg	128 ± 17	122 ± 14	132 ± 16	142 ± 19	<.001
Diastolic BP, mmHg	76 ± 10	75 ± 10	77 ± 10	76 ± 10	0.005
BMI <23 kg/m^2^	152 (15.8)	75 (14.8)	48 (17.9)	18 (13.3)	0.423
23 to <27.5	462 (48.2)	251 (49.5)	119 (44.4)	68 (50.4)	
≥27.5	345 (36.0)	181 (35.7)	101 (37.7)	49 (36.3)	
Waist circumference, cm	95.2 ± 10.4	93.5 ± 10.3	96.6 ± 10.0	98.9 ± 10.1	<.001
Hypertension†	486 (50.6)	147 (29.0)	173 (64.6)	128 (94.1)	<.001
Diabetes‡	374 (39.0)	219 (43.2)	112 (41.8)	33 (24.3)	<.001
Total Cholesterol, mg/dL	189 ± 39	195 ± 36	182 ± 38	180 ± 46	<.001
HDL cholesterol, mg/dL	51 ± 14	54 ± 15	49 ± 13	46 ± 12	<.001
LDL cholesterol, mg/dL	113 ± 34	117 ± 32	108 ± 33	107 ± 41	<.001
Triglycerides, mg/dL	130 ± 67	127 ± 68	132 ± 64	139 ± 69	0.159
Lp(a), mg/dL	18 (9-37)	19 (10-37)	18 (9-38)	16 (9-30)	0.219
Insulin, pmol/L	62.0 (44.0-95.0)	60.0 (42.0-83.0)	71.5 (47.0-109.5)	71.7 (44.0-104.0)	<.001
Hba1c, %	6.1 ± 0.9	5.8 ± 0.6	6.4 ± 1.0	6.7 ± 1.0	<.001
HOMA-IR	2.5 (1.7-4.0)	2.3 (1.6-3.3)	3.0 (1.9-4.7)	3.2 (2.0-5.1)	<.001
HOMA-BETA	99.9 (68.6-146.4)	106.2 (75.6-151.4)	99.8 (63.9-146.7)	77.9 (54.8-115.2)	0.045
Hepatic attenuation (HU)	54 ± 12	56 ± 11	51 ± 11	51 ± 12	<.001
Visceral fat area (cm^2^)	134 ± 55	120 ± 48	147 ± 54	165 ± 62	<.001
eGFR, mL/min/1.73 m^2^	90.9 ± 14.1	95.3 ± 11.6	88.6 ± 13.6	79.3 ± 16.0	<.001
Statin use	308 (32.1)	115 (22.7)	96 (35.8)	63 (46.3)	<.001
Hypertension medication use	369 (38.4)	95 (18.7)	132 (49.3)	106 (77.9)	<.001
CAC = 0	390 (40.6)	305 (60.2)	60 (22.4)	15 (11.0)	<.001
CAC 1–99	281 (29.3)	134 (26.4)	95 (35.4)	42 (30.9)	
CAC ≥100	289 (30.1)	68 (13.4)	113 (42.2)	79 (58.1)	
TAC = 0	444 (46.3)	339 (66.9)	76 (28.4)	16 (11.8)	<.001
TAC 1–99	287 (29.9)	132 (26.0)	107 (39.9)	39 (28.7)	
TAC ≥100	229 (23.9)	36 (7.1)	85 (31.7)	81 (59.6)	

BMI indicates body mass index; HDL, high-density lipoprotein; LDL, low-density lipoprotein; Lp(a), lipoprotein A; Hba1c, hemoglobin A1c; HOMA-IR, homeostatic assessment for insulin resistance; HU, Hounsfield units; eGFR, estimated glomerular filtration rate; CAC, coronary artery calcium; TAC, thoracic aortic calcification.

Values are presented as n (%), or mean ± SD, or median (interquartile range), as appropriate.

† Hypertension was defined as systolic blood pressure ≥140 mm Hg and/or diastolic blood pressure ≥90 mm Hg and/or use of any antihypertension medication.

‡ Diabetes mellitus was defined as fasting glucose ≥126 mg/dL and/or use of an anti-diabetes medication.

In Tobit regression analyses, older age, male, hypertension, diabetes, LDL-cholesterol, HOMA-IR, hepatic attenuation, visceral fat area, waist circumference, systolic blood pressure, eGFR, Hba1c, statin use, and hypertension medication use were associated with the presence of any TAC and any CAC (Table 2). Current smoking, HDL-cholesterol, triglycerides, diastolic blood pressure, and fasting insulin were associated with only CAC. There were no additional variables that were associated only with TAC; Lp(a) was not associated with either TAC or CAC. In Tobit regression models, hypertension, statin use, and hypertension medication use had the strongest relationships with TAC. Male gender, hypertension medication use, and hypertension had the strongest associations with CAC.

**Table 2:** Associations between risk factors and any TAC or CAC in MASALA.

	TAC		CAC	
Characteristics	Estimate (SE)	P value	Estimate (SE)	P value
Age, year	0.094 (0.006)	<0.001	0.072 (0.006)	<0.001
Men	0.301 (0.082)	<0.001	0.999 (0.086)	<0.001
Hypertension	0.806 (0.083)	<0.001	0.848 (0.085)	<0.001
Diabetes	0.546 (0.091)	<0.001	0.598 (0.094)	<0.001
Current smoking	0.048 (0.246)	0.845	0.676 (0.283)	0.017
HDL per SD	-0.062 (0.041)	0.128	-0.208 (0.041)	<0.001
LDL per SD	-0.179 (0.041)	<0.001	-0.184 (0.042)	<0.001
Triglycerides per SD	-0.042 (0.041)	0.298	0.097 (0.044)	0.026
Lp(a) per SD	-0.000 (0.041)	0.999	0.038 (0.042)	0.356
Insulin, per SD	0.081 (0.042)	0.055	0.134 (0.044)	0.002
HOMA-IR, per SD	0.113 (0.046)	0.015	0.144 (0.049)	0.003
HOMA-BETA, per SD	-0.052 (0.041)	0.203	-0.022 (0.042)	0.604
Hepatic attenuation (HU), per SD	-0.102 (0.041)	0.013	-0.174 (0.042)	<0.001
Visceral fat area (cm^2^), per SD	0.224 (0.049)	<0.001	0.303 (0.052)	<0.001
Asian BMI categories				
Normal (<23 kg/m^2^)	–	–	–	–
Overweight (23-27.4)	0.010 (0.117)	0.929	0.036 (0.118)	0.759
Obese (≥27.5)	0.153 (0.122)	0.210	0.054 (0.123)	0.660
Waist circumference (cm) per SD	0.201 (0.041)	<0.001	0.264 (0.043)	<0.001
Systolic blood pressure per SD	0.321 (0.044)	<0.001	0.308 (0.044)	<0.001
Diastolic blood pressure per SD	-0.001 (0.041)	0.980	0.106 (0.041)	0.010
eGFR per SD	-0.321 (0.043)	<0.001	-0.382 (0.046)	<0.001
Hba1c per SD	0.250 (0.044)	<0.001	0.244 (0.044)	<0.001
Statin use	0.749 (0.091)	<0.001	0.845 (0.095)	<0.001
Hypertension medication use	0.743 (0.086)	<0.001	0.885 (0.090)	<0.001

HDL indicates high-density lipoprotein; SD, standard deviation; LDL, low-density lipoprotein; Lp(a), lipoprotein A; HOMA-IR, homeostatic assessment for insulin resistance; HU, Hounsfield units; BMI, body mass index; eGFR, estimated glomerular filtration rate; Hba1c, Hemoglobin A1c

Of the 911 participants with PREVENT risk scores, the median PREVENT risk score was 4.3 %; 55.7 % of participants had a PREVENT score <5 %; 29.4 % had a PREVENT score 5 % to <10 % and 14.9 % had score ≥ 10 %; only 0.5 % of participants had scores ≥ 20 %. (Table 1). Participants with high (≥10 %) PREVENT scores were more likely to be older, male, have lived more years in the US, have higher systolic and diastolic blood pressure, total cholesterol, HDL and LDL cholesterol, waist circumference, Hba1c, fasting insulin, hypertension, diabetes, lower eGFR, and higher TAC and CAC scores.

**Fig. 1:**
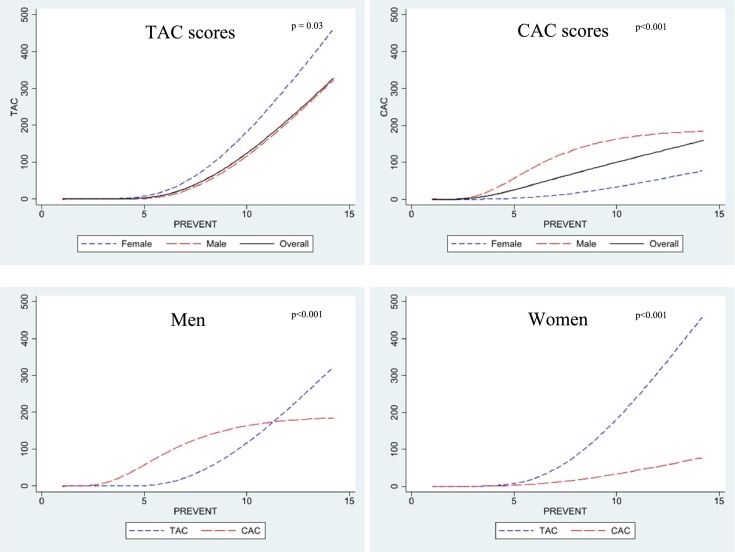
Restricted cubic spline plot of the association between PREVENT scores with TAC (top left) and CAC (top right) scores, overall and stratified by gender (men = bottom left; women = bottom right) using participant data in the 5^th^ to 95^th^ percentile of PREVENT score.

Restricted cubic spline plots determining associations between PREVENT risk estimates and TAC and CAC median scores revealed that with PREVENT scores ≥7 % women had higher TAC burden than men. Across PREVENT scores, men had higher CAC burden than women, though at very high PREVENT scores (>12 %), TAC was higher than CAC in men (Fig. 1).

**Fig. 2:**
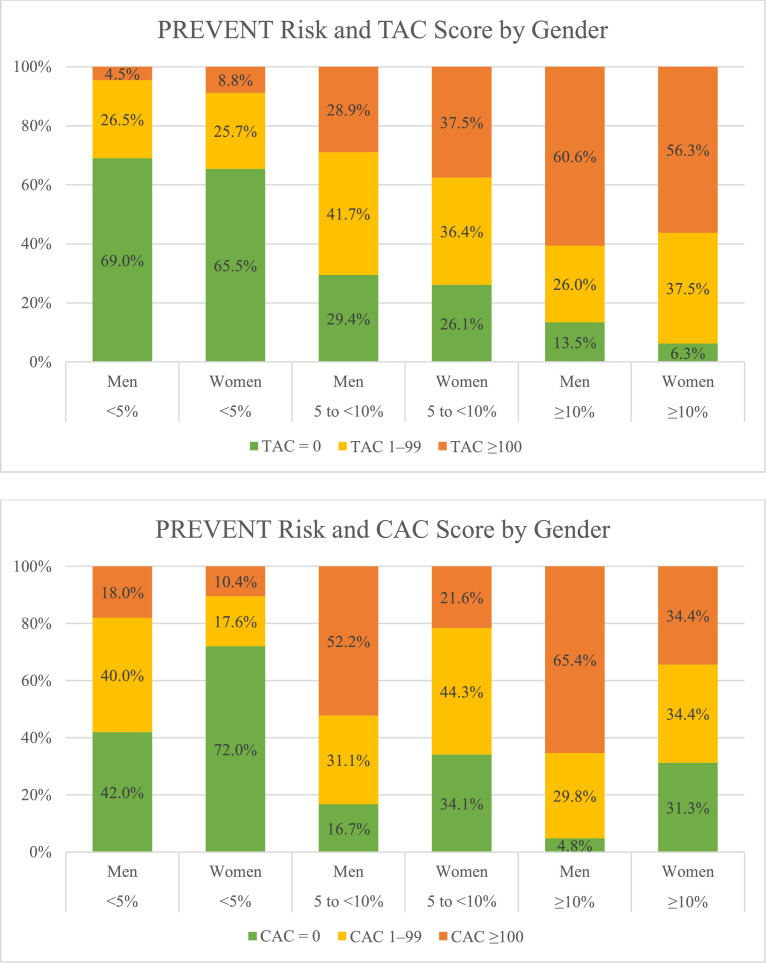
Distribution of TAC and CAC score categories (0, 1–99, ≥100) by PREVENT risk (<5 %, 5 to <10 %, ≥10 %) by gender.

There were important discordances for the association of PREVENT score category with TAC and CAC category (Fig. 2). Among individuals with PREVENT <5 %, there was more discordance with high CAC than with high TAC: 18.0 % of men and 10.4 % of women had CAC ≥100; while 4.5 % of men and 8.8 % of women had TAC ≥100. For those with PREVENT ≥10 %, the discordance with calcification varied by gender: 4.8 % of men and 31.3 % of women had no detectable CAC, whereas 13.5 % of men and 6.3 % of women had no detectable TAC. PREVENT scores between 5 and <10 % revealed similar frequency of TAC>0 between genders but more discordance with CAC>0 (83.3 % in men versus 65.9 % in women). Among those with CAC ≥100, 51.6 % of women and 18.2 % of men had PREVENT <5 % scores; among those with TAC ≥100, 34.6 % of women and 7.3 % of men had PREVENT <5 % scores.

## 4 Discussion

In the MASALA study population, a higher PREVENT score was associated with both higher TAC and CAC scores, but there were some important differences by gender. As PREVENT risk increased, women had higher TAC burden than men and across most PREVENT risk, CAC was higher in men than women. When examining concordance between PREVENT and calcification category, for participants in the 5 to <10 % or ≥10 % PREVENT categories, women were noted to have zero CAC and TAC>0 more frequently than men. These data suggest that TAC may be more useful in identifying subclinical atherosclerosis in women for possible reclassification of ASCVD risk.

This is the first study to identify TAC burden in South Asians. The TAC burden among MASALA participants was substantially higher than in the Multi-Ethnic Study of Atherosclerosis (MESA) despite the higher mean study population age in MESA. Approximately 54 % of participants in MASALA had any TAC versus 28 % in MESA overall (42 % in White, 38 % in Chinese, 30 % in Hispanic and 27 % in African Americans) [21,22]. TAC was seen in 29 % of women in MESA with important differences by age [21]: Among women in MESA younger than 65 years, non-Hispanic White individuals had the highest prevalence of TAC (19 %), followed by African American (17 %), and Chinese (16 %). In women in MESA ≥65 years, Chinese participants had highest prevalence of TAC (65 %), followed by non-Hispanic White (63 %) [22]. These data highlight a higher burden of TAC in South Asians compared to other U.S. population groups.

This study adds to a growing body of literature suggesting the utility of TAC in assessing ASCVD risk in women. Prior reports have shown an association between the presence of TAC and incident CVD events and all-cause mortality [10], and the addition of TAC >300 to CAC alone has been shown to improve risk reclassification in individuals at intermediate ASCVD risk by the PCE [11]. TAC has also been associated with stroke mortality independent of CAC and traditional risk factors in women more than in men [23], and in MESA, TAC ≥ 0 was a significant predictor of future coronary events independent of traditional risk factors in women only [24]. However, several other studies have demonstrated no increased prognostic value of TAC to prediction of cardiovascular disease and all-cause mortality after adjustment for risk factors [25,26]. Our study suggests that TAC may serve as a useful risk reclassification tool in women, especially given the high levels of discordance between PREVENT category and CAC score in men versus women.

To our knowledge, our study is the first to evaluate the relationship between PREVENT scores and measures of different regions of vascular calcification in South Asians. While less than 3 % of individuals in the initial derivation sample for PREVENT were reported to be Asians, emerging literature has explored the validity of PREVENT in South Asian populations. Yan et al. utilized the electronic health record to retrospectively examine the relationship between predicted ASCVD events by PREVENT and *International Classification of Diseases*-coded CVD events among disaggregated Asian subgroups. Asian Indians, comprising 31 % of the Asian population sample, had the highest C-statistic (0.85, 95 % CI 0.83-0.87) for total CVD, though PREVENT notably over-predicted risk for all CVD event subtypes in Indian subpopulations [27].

As the clinical application of PREVENT evolves, markers of subclinical atherosclerosis be even more important to stratify and reclassify individuals for better disease prevention. In this sample, 34.1 % of women with PREVENT ≥5 % were found to have CAC of 0, which has been demonstrated to be strongly correlated with the absence of ASCVD and low rates of incident cardiovascular events [28]. This is similar to a prior MASALA analysis which demonstrated South Asians who were categorized as low and intermediate risk by the PCE had higher odds of CAC = 0 in comparison to Non-Hispanic White counterparts.[29] While overestimation of risk is one possibility for these discordant findings, previous reports have demonstrated a positive correlation between PREVENT and CAC [30]. It is important to consider that the mean age of MASALA participants in this sample was less than 60 years, as South Asian women have been established to develop coronary artery calcium later in life compared to men [31,32]. While outcome data are needed to substantiate the ASCVD risk that PREVENT confers, our findings suggest there may be additional validity in TAC for younger South Asian women who potentially have high atherosclerotic risk without yet significant CAC burden. A smaller percentage (26.1 %) of women had PREVENT 5 to <10 % and TAC of 0. While the prognostic value of zero TAC is not yet known, if TAC of 0 was indeed demonstrated to have similar associations with decreased risk of ASCVD, TAC of 0 may aid in de-prescribing or avoiding unnecessary statin use in South Asian women.

## 5 Strengths and limitations

The cross-sectional nature of MASALA is a key limitation of this study. MASALA participants were recruited from two large metropolitan areas and may not be representative of all South Asians living in the United States. The sample of MASALA participants reflected in this analysis primarily consisted of participants of Indian descent, with relatively few Bangladeshi or Pakistani participants, who experience higher rates of ASCVD compared to individuals of Indian descent [33]. While identifying subclinical atherosclerosis is important for predictive purposes, TAC and CAC may not reflect the true morbidity and mortality of future ASCVD. ASCVD events data are not yet available for MASALA participants. Lastly, in MASALA, statin use was high, limiting application to a primary prevention population in which a statin decision is yet to be made. Future studies should examine the predictive utility of PREVENT in combination with subclinical markers of atherosclerosis for incident ASCVD events in South Asians.

## 6 Conclusions

In summary, in MASALA, higher category of PREVENT score was associated with higher TAC and CAC scores. Across most PREVENT scores, women had higher TAC scores than men and men had higher CAC scores than women. Among MASALA participants with PREVENT scores ≥5 %, women more frequently had zero CAC and TAC>0 than men. Future investigation should evaluate whether TAC is an appropriate tool for risk prognostication and reclassification of ASCVD risk prediction in South Asians, especially in women.

## 7 Ethics statement

Institutional review board committees at Northwestern University and the University of California, San Francisco approved study protocols and all participants gave written informed consent (IRB #10-00353).

## Funding

The project described was supported by Grant Number R01HL093009 from the National Heart, Lung, And Blood Institute and the National Center for Research Resources and the National Center for Advancing Translational Sciences, National Institutes of Health, through UCSF-CTSI Grant Number UL1RR024131 and UL1TR001872. The content is solely the responsibility of the authors and does not necessarily represent the official views of the National Heart, Lung, And Blood Institute or the 10.13039/100000002National Institutes of Health. The authors thank the other investigators, the staff, and the participants of the MASALA study for their valuable contributions.

**Figure:**
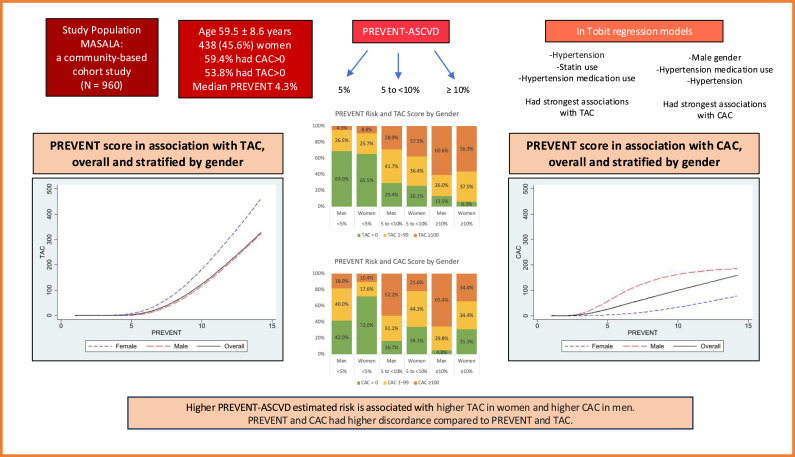
Central Illustration.

## Author agreement

The authors have agreed to the submission of this manuscript, February 2026, and the materials in this manuscript have not been previously published nor are in consideration for publication elsewhere.

## CRediT authorship contribution statement

**Eve M. Manghis:** Writing – review & editing, Writing – original draft, Conceptualization. **Rachel S. Chang:** Conceptualization. **Feng Lin:** Formal analysis, Conceptualization. **Meghana D. Gadgil:** Writing – review & editing, Conceptualization. **Nilay S. Shah:** Writing – review & editing, Conceptualization. **Matthew Budoff:** Writing – review & editing, Data curation, Conceptualization. **Alka M. Kanaya:** Writing – review & editing, Methodology, Data curation, Conceptualization.

## Declaration of competing interest

The authors declare that they have no known competing financial interests or personal relationships that could have appeared to influence the work reported in this paper.

## Appendix Supplementary materials

Image, application 1
