# Supplementary material for: Associations of the PREVENT Score with coronary artery and thoracic aortic calcification among South Asian Americans
Source: Am J Prev Cardiol. 2026 Mar 11;29:101538. doi: 10.1016/j.ajpc.2026.101538 (PMC13329547; doi:10.1016/j.ajpc.2026.101538)
Supplement: Supplementary file 1 [file mmc1.docx]

Supplemental Table 1. Percentage of MASALA sample with combinations of CAC and TAC prevalence, overall (black) and by gender (women = red, men = blue). P<0.001 for all analyses.

|  | Overall | |  | Women |  |  | Men |  |
| --- | --- | --- | --- | --- | --- | --- | --- | --- |
|  | TAC = 0 | TAC >0 |  | TAC = 0 | TAC >0 |  | TAC = 0 | TAC >0 |
| CAC = 0 | 29.27 | 11.35 | CAC = 0 | 43.61 | 17.35 | CAC = 0 | 17.24 | 6.32 |
| CAC >0 | 16.98 | 42.40 | CAC >0 | 9.13 | 29.91 | CAC >0 | 23.56 | 52.87 |

Supplemental Table 2. Mean and median CAC and TAC scores, Agatston units.

|  | PREVENT |  | Mean ± SD | Median (IQR) |
| --- | --- | --- | --- | --- |
| Overall | ≤5% | TAC | 29 ± 120 | 0 (0-6) |
|  |  | CAC | 58 ± 182 | 0 (0-36) |
|  | 5 to <10% | TAC | 216 ± 508 | 28 (0-161) |
|  |  | CAC | 231 ± 435 | 49 (3-283) |
|  | ≥10% | TAC | 755 ± 1624 | 253 (34-762) |
|  |  | CAC | 452 ± 670 | 175 (26-590) |
| Men | ≤5% | TAC | 17 ± 58 | 0 (0-4) |
|  |  | CAC | 86 ± 230 | 9 (0-74) |
|  | 5 to <10% | TAC | 197 ± 531 | 25 (0-126) |
|  |  | CAC | 295 ± 497 | 116 (9-368) |
|  | ≥10% | TAC | 668 ± 1610 | 257 (32-618) |
|  |  | CAC | 522 ± 717 | 216 (50-761) |
| Women | ≤5% | TAC | 36 ± 147 | 0 (0-10) |
|  |  | CAC | 40 ± 140 | 0 (0-3) |
|  | 5 to <10% | TAC | 255 ± 458 | 39 (0-237) |
|  |  | CAC | 101 ± 218 | 14 (0-86) |
|  | ≥10% | TAC | 1039 ± 1664 | 220 (34-1098) |
|  |  | CAC | 227 ± 423 | 43 (0-288) |
